# Deprivation of root-derived resources affects microbial biomass but not community structure in litter and soil

**DOI:** 10.1371/journal.pone.0214233

**Published:** 2019-03-28

**Authors:** Sarah L. Bluhm, Bernhard Eitzinger, Olga Ferlian, Christian Bluhm, Kristina Schröter, Rodica Pena, Mark Maraun, Stefan Scheu

**Affiliations:** 1 University of Göttingen, J.F. Blumenbach Institute of Zoology and Anthropology, Animal Ecology, Untere Karspüle 2, Göttingen, Germany; 2 University of Göttingen, Büsgen Institute, Forest Botany and Tree Physiology, Büsgenweg 2, Göttingen, Germany; 3 University of Göttingen, Centre of Biodiversity and Sustainable Land Use, Göttingen, Germany; Pacific Northwest National Laboratory, UNITED STATES

## Abstract

The input of plant leaf litter has been assumed to be the most important resource for soil organisms of forest ecosystems, but there is increasing evidence that root-derived resources may be more important. By trenching roots of trees in deciduous and coniferous forests, we cut-off the input of root-derived resources and investigated the response of microorganisms using substrate-induced respiration and phospholipid fatty acid (PLFA) analysis. After one and three years, root trenching strongly decreased microbial biomass and concentrations of PLFAs by about 20%, but the microbial community structure was little affected and the effects were similar in deciduous and coniferous forests. However, the reduction in microbial biomass varied between regions and was more pronounced in forests on limestone soils (Hainich) than in those on sandy soils (Schorfheide). Trenching also reduced microbial biomass in the litter layer but only in the Hainich after one year, whereas fungal and bacterial marker PLFAs as well as the fungal-to-plant marker ratio in litter were reduced in the Schorfheide both after one and three years. The pronounced differences between forests of the two regions suggest that root-derived resources are more important in fueling soil microorganisms of base-rich forests characterized by mull humus than in forests poor in base cations characterized by moder soils. The reduction in microbial biomass and changes in microbial community characteristics in the litter layer suggests that litter microorganisms do not exclusively rely on resources from decomposing litter but also from roots, i.e. from resources based on labile recently fixed carbon. Our results suggest that both bacteria and fungi heavily depend on root-derived resources with both suffering to a similar extent to deprivation of these resources. Further, the results indicate that the community structure of microorganisms is remarkably resistant to changes in resource supply and adapts quickly to new conditions irrespective of tree species composition and forest management.

## Introduction

Forests contribute to carbon sequestration by fixing carbon (C) in plant biomass as well as soil organic matter [1–3], but the amount of C sequestered depends on climate, soil and tree species [4–6]. In temperate forests soil C sequestration is driven by interactions between soil fertility, litter quality and decomposer organisms [7]. Further, disturbances due to forest management practices and plantation of different tree species [6,8–10] as well as forest age [11] result in shifts in C storage, and this has major implications for global C sequestration as most forests are used by humans.

Leaf litter has been assumed to be the largest pool of C entering forest soils, but it has been shown that roots and belowground inputs such as rhizodeposition may be more important [12–15]. Rhizodeposits consist of exudates, leakages, secretions, mucilages, mucigel and lysates [16,17] with the amount released depending on biotic and abiotic factors [16,18,19].

Depending on plant species and season, 10–63% of the photosynthetically fixed C is allocated to roots [20,21] and is actively or passively exuded into the soil [16]. The amount of rhizodeposits is closely related to fine root biomass [22–24] and varies with tree species and age, with the biomass in beech exceeding that in spruce [25] and reaching a peak at canopy closure after which it declines in maturing stands [26,27]. Exudates in the rhizosphere form microbial hotspots as the labile C compounds therein as well as nutrients from soil are processed quickly by microorganisms [28]. Thereby, the abundance of microorganisms in the rhizosphere is enhanced (‘rhizosphere effect’). Besides affecting the abundance of microorganisms, rhizodeposits also strongly impact the structure of microbial communities [29,30], but the effects are not yet fully understood. It has been assumed for long that mainly bacteria benefit from labile C in the rhizosphere [31,32], but this view has been challenged by recent studies documenting that also fungi benefit from labile C input and may exploit it even faster than bacteria [33–35]. This has major implications for the whole soil community as root C is incorporated into higher trophic levels of the soil food web via the fungal and bacterial energy channel [13,36,37]. By manipulating the input of rhizosphere resources their role in structuring microbial communities and energy channels in soil food webs as well as their effects on ecosystem functioning can be investigated. Root trenching, i.e. cutting off roots and preventing their regrowth, is a powerful tool to investigate these effects [38].

In this study, we investigated how the decrease in root-derived resources affects soil microbial biomass and community composition as indicated by substrate-induced respiration and phospholipid fatty acid analysis in forests of different management intensity, tree species composition and soil type. We hypothesized that (1) the reduction of root-derived resources affects both fungi and bacteria to a similar extent, and (2) the response of microorganisms to reduced input of root-derived resources correlates with the difference in fine root biomass between Hainich and Schorfheide, and (3) this response is more pronounced in deciduous forests as compared to coniferous, and also in young as compared to old deciduous forests.

## Materials and methods

### Study sites and experimental set-up

The experiment was established in two regions in Germany at sites of the “Biodiversity Exploratories”, a large-scale and long-term biodiversity project (www.biodiversity-exploratories.de, Fischer et al. 2010). The regions are Schorfheide-Chorin (hereafter *Schorfheide*), located in the lowlands (3–140 m a.s.l.) of Northeastern Germany and characterized by postglacial geomorphological structures, and the Hainich-Dün (hereafter *Hainich*), situated in the uplands (285–550 m a.s.l.) of Central Germany. Mean annual temperatures at Schorfheide and Hainich are 8.0–8.5 and 6.5–8.0°C with mean annual precipitation of 500–600 and 500–800 mm, respectively. The soil are mainly Cambisols in the Schorfheide and Luvisols in the Hainich with soil pH averaging 3.00 ± 0.19 and 4.59 ± 0.67, respectively. Bedrock is glacial till in the Schorfheide and Triassic limestone in the Hainich. For more details on the study sites see [39] and [40]. In each of the two regions, we selected 16 forest sites representing four different forest types: (1) managed coniferous forests with Norway spruce *Picea abies* in the Hainich (‘spruce’) and Scots pine *Pinus sylvestris* in the Schorfheide (‘pine’), (2) 30 years old managed beech forests (‘young beech’), (3) 70 years old managed beech forests (‘old beech’), and (4) unmanaged natural beech forests left out of management for at least 60 years with some trees being 120 to 150 years old (‘unmanaged beech’). All plots were randomly arranged with the minimum distance between sites being 500 m. Beech forests are dominated by *Fagus sylvatica*, interspersed with ash, *Fraxinus excelsior*, and sycamore, *Acer pseudoplatanus*.

### Establishment and maintenance of root-trenching plots

Between September and October 2011, in each of the selected 32 sites in Hainich and Schorfheide, one trenched and one control subplot was established. For the trenched subplots trenches were cut into soil along the perimeter of an area of 120 × 120 cm to a depth of 40–50 cm using a chainsaw, thereby cutting all tree roots. The bulk of fine tree roots at our study sites are found in the uppermost 20 cm of soil [41]. However, to more fully exclude roots we decided to cut trenches to a depth of 50 cm where bedrock prevents further root growth. To prevent re-colonization of the plots by tree roots, trenches were stabilized by inserting polyethylene barriers (120 × 60 × 0.5 cm) on each of the four sides of the subplots. In addition, we inserted aluminum linings at the corners to close the gap between adjacent barriers.

Polyethylene barriers extended ca. 10 cm aboveground to restrict colonization of the plots by animals from outside. To control for potential side effects of aboveground parts of the barriers, control plots, which were established in close vicinity of the treatment plots, were also equipped with respective barriers aboveground.

Herbaceous plants and grasses in root-trenching and control plots were clipped at regular intervals during the growth period to minimize input of root-derived carbon. Soil moisture was measured gravimetrically from soil cores (10 cm deep, 5 cm diameter) taken in July and August every year in each treatment and control plots to check for differences in soil water content. To control for differences in water content (soil water content in trenched plots was on average 5% higher) we added water to control plots equalizing the amount of water in the upper 10 cm of the soil in control and trenched plots.

### Abundance of mycorrhized root tips and fine root biomass

In May 2013, soil cores (20 cm deep, 8 cm in diameter) were taken from each of the plots and transferred to the laboratory. The soil was soaked with water and the roots were carefully washed. Adherent soil was removed and roots were kept moist in wet tissue paper at 4°C. Roots of other plant species than ectomycorrhizal trees, mainly *Acer* sp., *Fraxinus excelsior* and herbal plants, were generally rare and not considered further. The root tips of spruce, pine and beech were inspected using a binocular (Leica DFC 420C, Wetzlar, Germany). From each sample a maximum of 500 root tips were counted and classified as dead and vital tips for calculating the ratio between dead and vital root tips. Vital root tips were further divided into mycorrhized (with hyphal mantle) or non-mycorrhized (without hyphal mantle, white and thin). Mycorrhization rate was calculated according to [42].

### Microbial biomass and community composition

One and three years after experimental set-up, i.e. in October 2012 and 2014, we analyzed microbial biomass (C_mic_) and microbial community composition in litter and soil of each plot. For measurement, we pooled each litter (L layer) and soil of three soil cores (5 cm deep, 5 cm diameter) taken from each of the plots. Soil material predominantly comprised mineral soil (Ah layer), but in some plots also included F- and H-material. Soil samples were sieved through 2 mm mesh and litter samples were cut into small pieces (< 2 mm) using scissors and then stored at -20°C.

C_mic_ in litter and soil was determined using substrate-induced respiration (SIR), i.e. the respiratory response of microorganisms after glucose addition [43]. Respiration rates were measured using an automated O_2_ microcompensation system after supplementing samples with 8 or 80 mg glucose g^−1^ dry weight (DW) for soil or litter, respectively [44,45]; the mean of the three lowest values within 2–10 h after addition of glucose was taken as the maximum initial respiratory response (MIRR). C_mic_ (μg C g^−1^ soil or litter DW) was calculated as 38×MIRR [46].

To track changes in soil microbial community, phospholipid fatty acids (PLFA) were examined. Although less powerful than DNA and RNA based approaches in analyzing the phenotype and activity of microbial communities PLFA analysis provides broadly comparable results on the structure of microbial communities [47]. PLFAs in soil were measured from samples taken in 2012 and 2014, whereas PLFAs in litter were only measured from samples taken in 2014. For PLFA analysis 2 g of leaf litter and 4 g of soil (wet weight) were extracted following the protocol of [48] and subsequently identified following [49]. PLFA abundances were calculated as nmol per gram dry weight of soil or leaf litter. The PLFA 18:2ω6,9 was used as fungal biomarker, 18:1ω9 as plant marker and the following FAs served as biomarkers for bacteria: i15:0, a15:0, i16:0 and i17:0 (Gram-positive bacteria), cy17:0 and cy19:0 (Gram-negative bacteria), and 16:1ω7 and 18:1ω7 [50,51]. The ratio between 18:2ω6,9 and 18:1ω9 was used as a measure of the fungal-to-plant ratio and the ratio between 18:2ω6,9 and the sum of bacterial PLFAs as a measure of the fungal-to-bacterial ratio. The ratios of the sum of cyclopropyl PLFAs to their monoenoic precursors [(cy17:0 + cy19:0)/(16:1ω7 + 18:1ω7); cy-to-pre ratio], and of saturated (SAT) and monounsaturated (MONO) PLFAs were used as microbial stress indicators [52].

### Statistical analyses

Statistical analyses were performed using R v 3.4.3 [53] and Statistica Version 12 (Dell Inc. 2015). Marker PLFAs were analyzed separately for soil and litter. The effect of root trenching (control and trenched plots), time after root trenching (one and three years), region (Hainich and Schorfheide), forest type (young, old and unmanaged beech forests, coniferous forests) and their interactions on C_mic_ and marker PLFAs were analyzed using linear mixed effects models implemented in the R package ‘nlme’ v 3.1–131 [54]. A random effect of sampling date (year) within plot (plot ID) was included to account for the split-plot design of control and trenched plots and repeated measurements within forest sites. Region was used as fixed factor. Differences between means were inspected using Tukey’s honestly significant difference (HSD) test at P < 0.05. Microbial biomass and marker FAs were log-transformed prior to the analyses. PLFA concentrations and microbial biomass were log-transformed.

In the linear mixed effects models for analyzing C_mic_ in litter and soil, soil water content was included as covariable. The effects of root trenching on PLFA profiles were analyzed using non-metric multidimensional scaling (NMDS) implemented in the R package ‘vegan’ v 2.4–3 [54] followed by discriminant function analysis (DFA) using Statistica Version 12 (Dell Inc. 2015). Data provided in the text represent means and standard deviations.

## Results

### Roots

After 1.5 years root trenching significantly reduced vital root tips from 44.3 ± 11.6 to 18.3 ± 20.7% in Hainich and from 37.5 ±14.3 to 19.4 ± 15.7% in Schorfheide (F_1,12_ = 39.15, p < 0.001 and F_1,12_ = 14.29, p = 0.003, respectively). Root trenching also significantly reduced ectomycorrhizal fine root biomass from 1.98 ± 0.8 to 0.9 ± 0.6 g fresh weight in Hainich and from 2.9 ± 2.7 to 1.6 ± 1.5 g fresh weight in Schorfheide (F_1,12_ = 30.51, p < 0.001 and F_1,12_ = 11.56, p = 0.005, respectively).

### Microbial biomass

Trenching significantly reduced C_mic_ in soil after one and three years, but this depended on region. In Hainich C_mic_ significantly decreased from 991.5 ± 559.1 to 731.4 ± 441.2 μg C g^-1^ DW averaged over both years, while in Schorfheide C_mic_ decreased, but only marginally significant from 292.9 ± 201.4 to 262.2 ± 189.9 μg C g^-1^ DW (Table 1). Trenching also significantly decreased C_mic_ in the litter layer, but only after one year in Hainich from 9724 ± 3435 to 7536 ± 3246 μg C g^-1^ DW (significant trenching × year interaction), while in Schorfheide trenching did not affect C_mic_ in the litter layer (Table 1).

**Table 1:** LME table of F- and p-values. LME table of F- and p-values on the effect of root trenching (control and trenched plots), duration of root trenching (one and three years) and forest type (young beech, old beech, unmanaged beech, coniferous) on microbial biomass (C_mic_), bacterial, Gram-negative, Gram-positive, fungal, plant marker PLFAs and fungal-to-bacterial, fungal-to-plant, cyclopropyl-to-monoenoic precursor PLFA ratio (cy-to-pre) and saturated-to-mono-unsaturated PLFA ratio (sat-to-mono) in litter and soil in Hainich and Schorfheide. Significant effects are highlighted in bold.

**Litter**	trenching		forest type		year		trenching × forest type		trenching × year		year × forest type	
**Hainich**	*F*	*p*	*F*	*p*	*F*	*p*	*F*	*p*	*F*	*p*	*F*	*p*
C_mic_	7.80	**0.011**	0.74	0.547	3.74	*0*.*077*	0.79	0.516	8.45	**0.009**	0.55	0.656
bacteria	1.16	0.302	1.88	0.187	nd	nd	0.04	0.989	nd	nd	nd	nd
Gram-negative	0.42	0.527	1.39	0.294	nd	nd	0.17	0.915	nd	nd	nd	nd
Gram-positive	1.18	0.299	2.57	0.103	nd	nd	0.14	0.935	nd	nd	nd	nd
fungal marker	0.03	0.856	0.30	0.826	nd	nd	1.03	0.412	nd	nd	nd	nd
plant marker	0.08	0.778	1.61	0.240	nd	nd	0.83	0.503	nd	nd	nd	nd
fungal-to-plant	1.33	0.271	0.61	0.620	nd	nd	1.28	0.327	nd	nd	nd	nd
fungal-to-bacterial	6.61	**0.024**	0.69	0.576	nd	nd	4.25	**0.029**	nd	nd	nd	nd
cy-to-pre	2.18	0.166	0.52	0.675	nd	nd	4.11	**0.032**	nd	nd	nd	nd
sat-to-mono	3.00	0.109	0.60	0.625	nd	nd	5.07	**0.017**	nd	nd	nd	nd
**Schorfheide**		** **		** **		** **		** **		** **		** **
C_mic_	0.73	0.403	3.98	**0.035**	1.39	0.262	0.71	0.555	0.04	0.848	1.25	0.335
bacteria	6.89	**0.022**	2.82	*0*.*084*	nd	nd	2.19	0.143	nd	nd	nd	nd
Gram-negative	5.82	**0.033**	2.87	*0*.*080*	nd	nd	1.95	0.175	nd	nd	nd	nd
Gram-positive	6.77	**0.023**	2.21	0.139	nd	nd	2.35	0.124	nd	nd	nd	nd
fungal marker	6.70	**0.024**	3.33	*0*.*056*	nd	nd	1.55	0.252	nd	nd	nd	nd
plant marker	6.34	**0.027**	2.41	0.118	nd	nd	2.11	0.152	nd	nd	nd	nd
fungal-to-plant	4.73	**0.050**	3.56	**0.048**	nd	nd	1.28	0.324	nd	nd	nd	nd
fungal-to-bacterial	0.50	0.492	1.60	0.237	nd	nd	0.30	0.818	nd	nd	nd	nd
cy-to-pre	1.12	0.312	3.34	*0*.*056*	nd	nd	0.10	0.960	nd	nd	nd	nd
sat-to-mono	4.25	*0*.*062*	5.15	**0.016**	nd	nd	1.07	0.398	nd	nd	nd	nd
**Soil**	trenching		forest type		year		trenching × forest type		trenching × year		year × forest type	
**Hainich**	*F*	*p*	*F*	*p*	*F*	*p*	*F*	*p*	*F*	*p*	*F*	*p*
C_mic_	10.88	**0.003**	0.97	0.440	13.86	**0.003**	0.60	0.620	0.01	0.930	0.24	0.864
bacteria	11.89	**0.002**	0.35	0.789	7.48	**0.018**	3.55	**0.028**	14.87	**0.001**	0.62	0.617
Gram-negative	11.18	**0.002**	1.34	0.306	5.51	**0.037**	4.44	**0.012**	16.33	**<0.001**	0.14	0.933
Gram-positive	10.97	**0.003**	0.31	0.819	11.13	**0.006**	3.06	**0.045**	16.57	**<0.001**	0.58	0.639
fungal marker	23.80	**<0.001**	0.11	0.954	0.82	0.383	2.59	*0*.*073*	15.99	**<0.001**	0.76	0.539
plant marker	11.08	**0.003**	0.10	0.957	4.17	*0*.*064*	2.83	*0*.*057*	12.52	**0.001**	0.90	0.472
fungal-to-plant	8.92	**0.006**	1.61	0.238	2.34	0.152	0.77	0.522	2.44	0.130	0.33	0.805
fungal-to-bacterial	7.37	**0.011**	2.37	0.122	8.93	**0.011**	0.83	0.488	2.20	0.150	0.27	0.844
cy-to-pre	0.66	0.423	0.32	0.814	5.42	**0.038**	0.39	0.762	0.08	0.783	0.35	0.788
sat-to-mono	6.21	**0.019**	0.19	0.904	18.26	**0.001**	1.08	0.376	1.14	0.294	0.43	0.735
**Schorfheide**		** **		** **		** **		** **		** **		
C_mic_	2.99	*0*.*098*	4.26	**0.029**	0.33	0.579	0.51	0.680	0.48	0.498	0.42	0.743
bacteria	18.08	**<0.001**	1.73	0.214	14.02	**0.003**	0.82	0.492	0.93	0.342	0.49	0.696
Gram-negative	12.49	**0.001**	1.26	0.332	5.55	**0.036**	0.40	0.757	0.74	0.396	0.06	0.978
Gram-positive	18.10	**<0.001**	0.79	0.520	14.82	**0.002**	1.26	0.308	0.76	0.391	0.76	0.539
fungal marker	18.45	**<0.001**	1.86	0.190	16.33	**0.002**	1.08	0.374	0.37	0.546	0.46	0.714
plant marker	16.72	**<0.001**	1.97	0.172	8.59	**0.013**	1.34	0.284	0.02	0.899	0.46	0.713
fungal-to-plant	4.27	**0.049**	1.91	0.181	3.68	*0*.*079*	0.17	0.919	1.09	0.305	0.01	0.997
fungal-to-bacterial	2.78	0.107	0.46	0.715	0.13	0.724	0.21	0.889	1.55	0.224	0.15	0.925
cy-to-pre	1.07	0.311	1.54	0.255	2.78	0.121	0.05	0.985	1.26	0.271	0.96	0.442
sat-to-mono	0.21	0.651	2.81	*0*.*084*	0.18	0.676	0.26	0.852	0.86	0.362	0.35	0.787

### Microbial community structure

**Fig 1:**
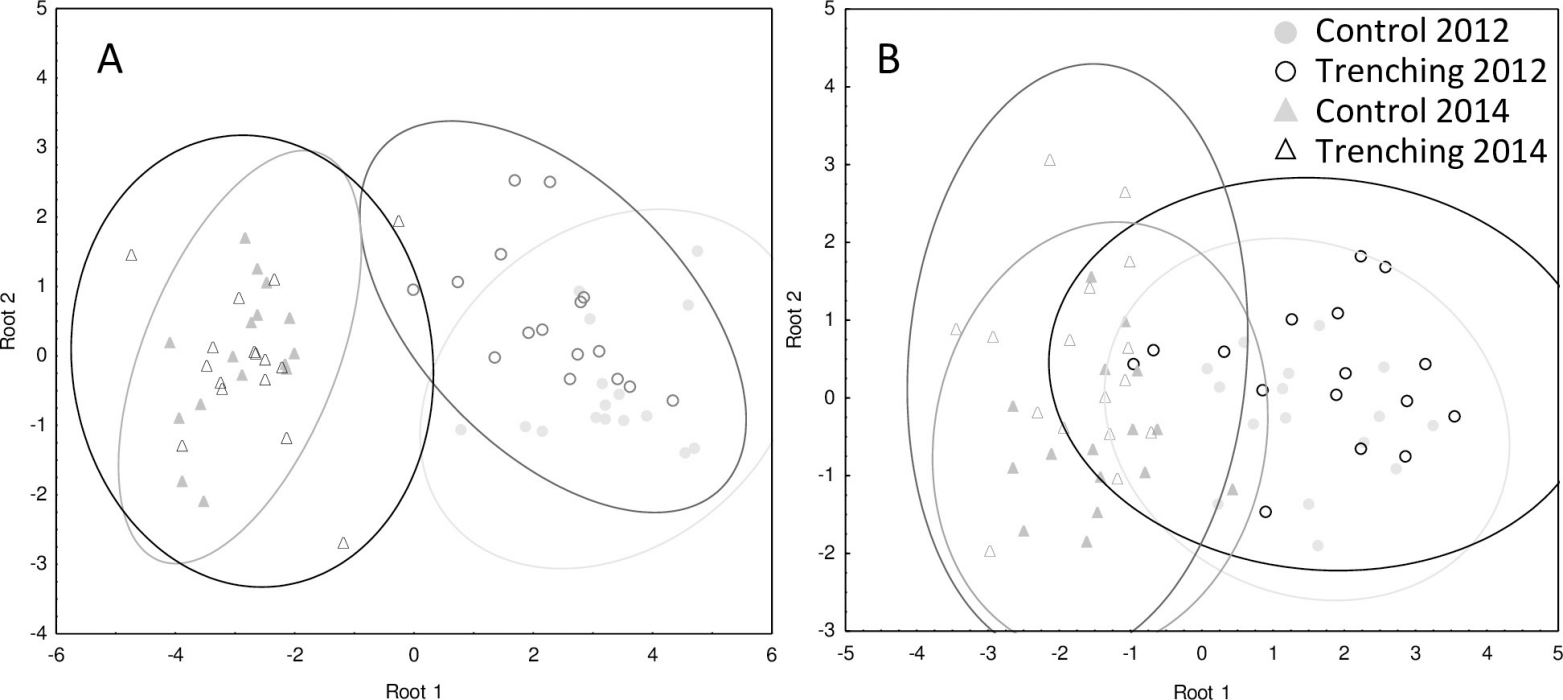
Discriminant function analysis. Discriminant function analysis of the PLFA composition of soil in control (Control) and root trenched plots (Trench) in Hainich (A) and Schorfheide (B) after one and three years of root trenching. Ellipses represent confidence intervals at 95%.

As indicated by PLFA patterns, microbial community structure in soil in the Hainich significantly differed between trenched and control plots, but this depended on year (Wilks Lambda 0.0906, F_15,154_ = 14.33, p < 0.001 for trenching × date) with the structure being significantly different between root trenched and control plots after one year but not after three years (squared mahalanobis distances (SMD) = 2.013, F = 3.01, p = 0.018 and SMD = 0.085, F = 0.13, p = 0.986, respectively, Fig 1A). By contrast, microbial community composition in soil in the Schorfheide was not affected by root trenching (Fig 1B). Neither in the Hainich nor in the Schorfheide microbial community structure in litter differed significantly between trenched and control plots after three years (Wilks Lambda 0.936, F_5,26_ = 0.36, p = 0.8 7and Wilks Lambda 0.883, F_3,28_ = 1.23, p = 0.32, respectively).

### Marker PLFAs

**Fig 2:**
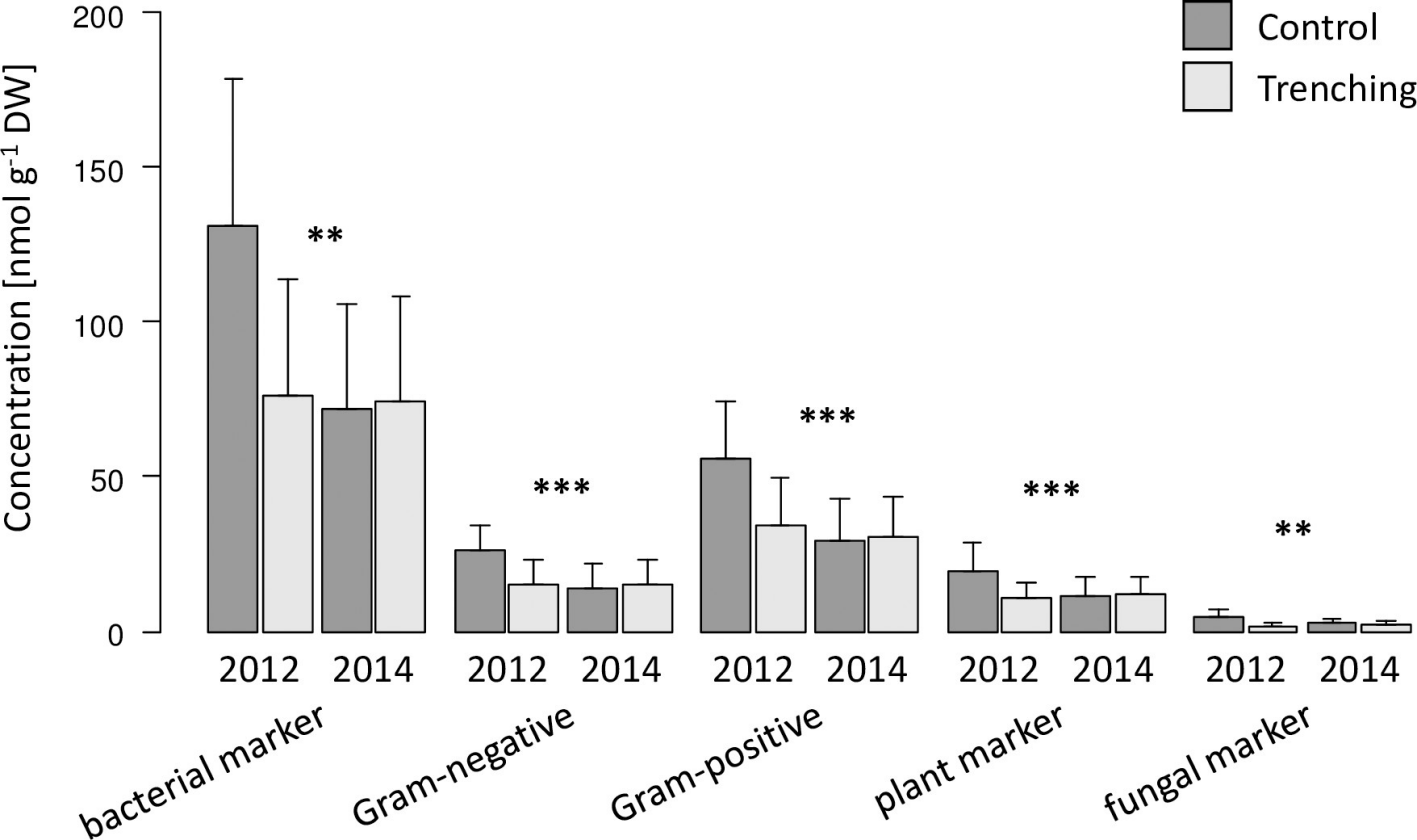
Marker PLFAs in soil in Hainich. Effect of root trenching on marker PLFAs (bacterial, Gram-negative, Gram-positive, plant and fungal) in soil in Hainich after one (2012) and three (2014) years after root trenching (pooled for forest type); *, p < 0.05; **, p < 0.01; ***, p < 0.001.

In Hainich, the sum of bacterial, Gram-negative and Gram-positive, plant (18:1ω9) and fungal (18:2ω6,9) marker PLFAs in soil were significantly reduced by root trenching, however, the effects varied with forest type and between years (significant trenching x forest type and trenching x year interaction; Table 1). With the exception of the fungal marker, which also was reduced in young beech forests, the reductions were restricted to unmanaged natural beech (Table 2). Further, the reductions generally were only present one year, but not three years after trenching (Fig 2). In addition to marker PLFAs, the fungal-to-bacterial ratio as well as the fungal-to-plant ratio averaged across both sampling dates decreased from 0.04 ± 0.01 to 0.03 ± 0.01 and from 0.24 ± 0.09 to 0.20 ± 0.05 in trenched plots, whereas the stress indicator SAT-to-MONO PLFA ratio increased from 2.01 ± 0.46 to 2.18 ± 0.53 in trenched plots (Table 1).

**Table 2:** Tukey test. Differences in marker PLFAs (bacterial, Gram-negative, Gram-positive, plant and fungal) between control (C) and trenched plots (T) in the four forest types as indicated by t-ratio and p-values of Tukey’s HSD tests. Significant differences are highlighted in bold.

	Beech old (C-T)		Beech young (C-T)		Beech natural (C-T)		Conif (C-T)	
	t-ratio	p-value	t-ratio	p-value	t-ratio	p-value	t-ratio	p-value
bacteria	1.26	0.906	2.44	0.262	3.83	**0.014**	-0.64	0.998
Gram-negative	0.76	0.994	2.26	0.352	4.36	**0.004**	-0.70	0.996
Gram-positive	1.27	0.902	2.36	0.299	3.56	**0.026**	-0.60	0.999
plant	1.04	0.963	2.82	0.132	3.30	**0.047**	-0.50	1.000
fungal	2.16	0.407	3.52	**0.029**	3.83	**0.014**	-0.19	1.000

**Fig 3:**
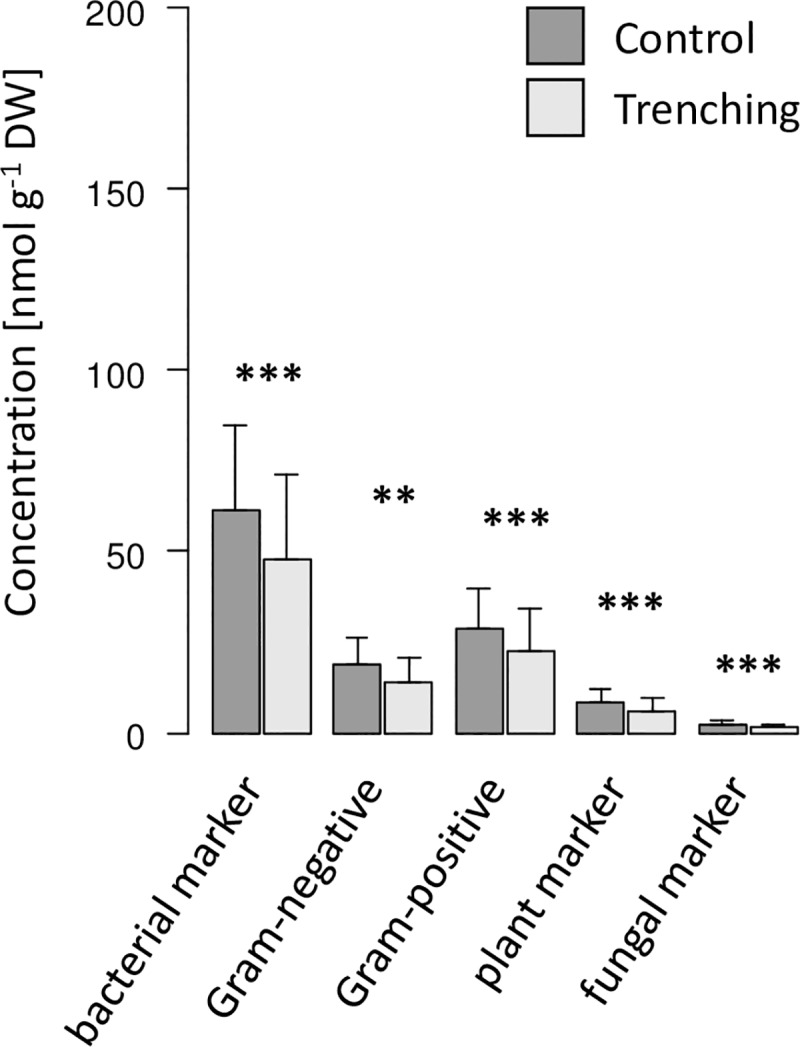
Marker PLFAs in soil in Schorfheide. Effect of root trenching on marker PLFAs (bacterial, Gram-negative, Gram-positive, plant and fungal) in soil in Schorfheide; means of samples taken in 2012 and 2014 (pooled for forest type); *, p < 0.05; **, p < 0.01; ***, p < 0.001.

In Schorfheide the total amount of PLFAs as well as the sum of bacterial, Gram-negative and Gram-positive, plant (18:1ω9) and fungal (18:2ω6,9) marker PLFAs in soil were significantly reduced by root trenching irrespective of the year of sampling (Table 1, Fig 3). By contrast, the fungal-to-bacterial PLFA ratio, fungal-to-plant PLFA ratio and the stress indicators cy-to-pre PLFA ratio and SAT-to-MONO PLFA ratio were not significantly affected by root trenching (Table 1).

**Fig 4:**
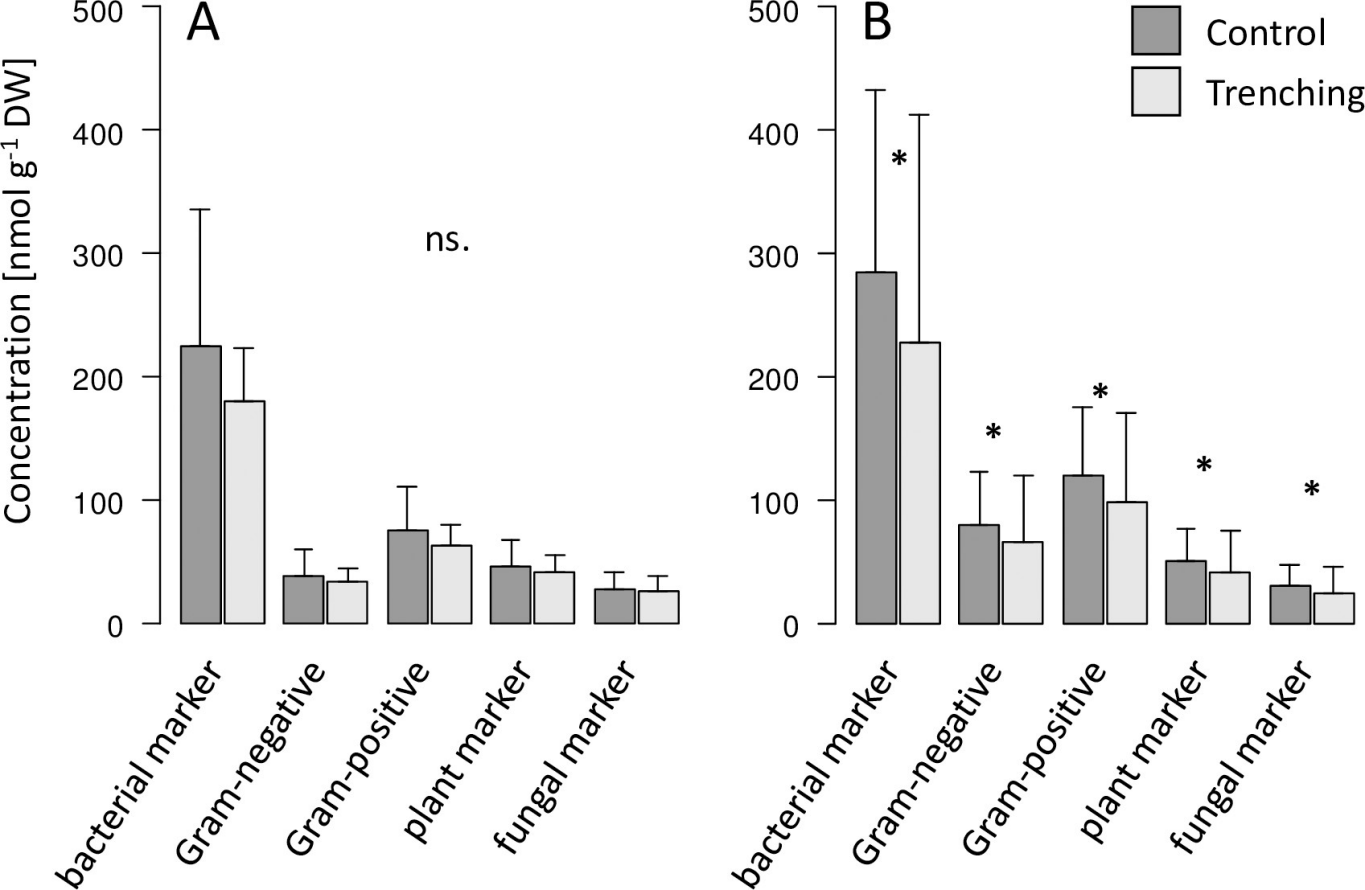
Marker PLFAs in litter in Hainich and Schorfheide. Effect of root trenching in litter in Hainich (A) and Schorfheide (B) after three (2014) years of root trenching on marker PLFAs (bacterial, Gram-negative, Gram-positive, plant and fungal); *, p < 0.05; **, p < 0.01; ***, p < 0.001.

PLFAs in litter were only determined in 2014. Compared to soil, trenching significantly increased the fungal-to-bacterial PLFA ratio in litter in the Hainich from 0.12 ± 0.03 to 0.15 ± 0.06 (Table 1). The same was true for the stress indicators cy-to-pre PLFA ratio and SAT-to-MONO PLFA ratio (Table 1), but this depended on forest type; differences were only significant in young beech stands where the stress indicators increased from 0.35 ± 0.21 to 0.52 ± 0.18 and from 1.45 ± 0.42 to 1.94 ± 0.28 for cy-to-pre and SAT-to-MONO, respectively (Tukey’s HSD test p = 0.051 and p = 0.034, respectively). In litter of the Schorfheide the sum of bacterial, Gram-negative and Gram-positive, plant (18:1ω9) and fungal (18:2ω6,9) marker PLFAs significantly decreased by trenching (Table 1, Fig 4). Also, trenching decreased the fungal-to-plant PLFA ratio from 0.58 ± 0.09 to 0.53 ± 0.13 in litter.

## Discussion

Results of the present study showed that microbial communities of forest soils heavily rely on the input of resources from tree roots. However, the response to deprivation of root-derived resources varied between forest types and the duration of root trenching. Remarkably, the results suggest that microorganisms in both litter and soil rely on root-derived resources with bacteria and fungi suffering to a similar extent from root trenching.

### Microbial biomass

Root trenching reduced vital fine roots by more than 50% after three years which is in line with Díaz-Pinés [38] who found a reduction of 30% after 1.5 years. Microbial biomass in root-trenched plots decreased in litter and soil, emphasizing that forest soil microorganisms of both litter and soil heavily rely on root-derived resources. Notably, however, the reduction in microbial biomass in soil of forests in Hainich was more pronounced than that in Schorfheide, suggesting that the importance of root-derived resources varies between base-rich forests characterized by mull humus (Hainich) and of forests poor in base cations characterized by moder soils (Schorfheide). Differences in pH and humus form are associated with differences in the biomass of roots and ectomycorrhizal fungi which are higher in Hainich as compared to Schorfheide [55,56]. As the biomass of fine roots and the amount of root exudates typically are correlated [22,23], this may explain the more pronounced impact of trenching in soil of forests of Hainich, corroborating our hypothesis.

In Hainich, root trenching only affected microbial biomass in the litter layer after one year but not after three years indicating high resilience after disturbances, as has been suggested earlier [57]. Notably, microbial biomass in control plots markedly differed between sampling dates, indicating that it heavily depends on annual weather conditions. Compared to the first sampling date in 2012, microbial biomass in control plots was much lower at the second sampling in 2014, but this did not corroborate with dryer conditions in 2014 as compared to 2012, indicating that other factors than soil moisture were responsible for the inter-annual differences in microbial biomass. Further, decomposition of roots cut by trenching and increased N availability via the absence of mycorrhiza may have mitigated the detrimental effects of trenching after three years, but presumably not after one year as roots decompose slowly [38,58].

### Microbial community structure

Despite the pronounced changes in microbial biomass, root trenching only little affected the community structure of microorganisms as indicated by PLFA analysis. Microbial community structure was only affected by root-trenching in soil in Hainich after one year, indicating that microbial communities recover quickly even after strong disturbances. It has been suggested earlier that old growth forest soils are buffered against disturbances such as trenching [57]. Reduced input of root-derived resources due to trenching might be compensated by an increased use of alternative resources such as decomposing roots cut by root trenching. Interlinkage of soil energy channels, such as those based on root-derived resources and resources from decomposing organic matter as recently investigated in pulse labelling experiments [33,59], likely contributed to the low responsiveness of microbial community structure in soil.

Microbial marker PLFAs generally were reduced by trenching in both Hainich and Schorfheide. Interestingly, root-trenching did not only affect microorganisms in soil, but also in the litter layer. This indicates that litter microorganisms do not exclusively rely on resources from decomposing litter, but also from roots, i.e. from resources based on freshly fixed carbon. However, the response of litter microorganisms varied between Hainich and Schorfheide with fungal and bacterial marker PLFAs as well as the fungal-to-plant marker ratio being only reduced in Schorfheide. Schorfheide is characterized by sandy soils with thick organic layers resulting from slow decomposition processes due to low soil pH. Notably, it has been shown that most root tips in the litter of Schorfheide occur in the organic layer [60,61], explaining the strong effect of root shortage on microbial markers. This contrasts the view that thick organic layers reduce access of soil biota to root-derived resources [40].

In soil, fungal and bacterial marker PLFAs were reduced by root trenching in both regions. In Hainich, root trenching reduced fungal and bacterial marker PLFAs by 60 and 40% after one year of root trenching respectively, but not after three years. The strong reduction after one year indicates that in Hainich microorganisms heavily rely on root-derived resources. Much higher microbial biomass and microbial marker PLFAs one year after root trenching than three years after root trenching suggests that trenching more strongly affects microorganisms if microorganisms are thriving, i.e. at favorable environmental conditions. In Schorfheide, fungal and bacterial marker PLFAs, including those for Gram-negative and Gram-positive bacteria, declined by 35 and 22%, respectively, with the reductions being similar in both years. The strong negative effects of trenching on microbial biomass as well as bacterial and fungal marker PLFAs are consistent with earlier findings [62], and suggest that microorganisms in soil of temperate deciduous forests stocking on sandy soil and being characterized by thick organic layers also heavily rely on root-derived resources. More consistent reduction in microbial biomass in Schorfheide across years as compared to Hainich indicates that soil microorganisms in the former more consistently rely on root-derived resources and are less susceptible to weather-induced changes in their response to these resources. The consistent reduction in the fungal marker PLFA likely reflects the reduced biomass of mycorrhizal fungi in trenched plots as observed previously [63].

In Hainich, trenching resulted in an increase in the SAT-to-MONO ratio in both soil and litter, indicating that Gram-negative bacteria were experiencing increased stress conditions and responded by changing their membrane lipids [52], potentially due to changes in nutrient supply [64]. Neither microbial biomass nor individual marker PLFAs were affected by forest management; this is consistent with earlier findings on the response of bacteria to forest management at our study sites [65].

## Conclusions

The results document that microorganisms in both litter and soil heavily rely on root-derived resources with bacteria and fungi responding in a similar way. This suggests that both to a similar extent rely on root-derived resources. By contrast, the response of microorganisms varied between regions, i.e. forests stocking on different parent rock and exposed to different climatic conditions, but was remarkably consistent across forest types. The variable response of microorganisms between years due to root trenching in Hainich suggests that under favorable conditions, where microbial biomass is high, shortage of nutrients has stronger impacts. The observed dependency of soil microbial communities on root exudates is likely to be affected by reduced soil moisture due climate change. The prospected increase in the length of drought events in central Europe [66,67] is likely to result in an increase in rhizodeposits [68], which, however, might be less pronounced if droughts persist for long [69]. The strong connection between soil microorganisms and tree roots therefore, at least in part, may offset negative impacts due to reduced soil water availability, explaining the microbial resilience observed in drought experiments [70]. Overall, however, the results indicate that the community composition of microorganisms in forest soils is highly resistant against changes in the input of root-derived resources.

## Supporting information

S1 Table Microbial biomass data. Microbial biomass (cmic) [μg C_mic_ g^-1^ soil or litter DW], basal respiration (bas) [μg O_2_ g^-1^ soil or litter DW h^-1^], substrate induced respiration (sir) [μg O_2_ h^-1^ g soil or litter DW^-1^] and gravimetrical water content (gravwater) as affected by region (Hainich-Dün and Schorfheide-Chorin), forest type (young, old and unmanaged beech forests, coniferous forests), treatment (trenched and control subplots) and layer (litter and soil) at the two sampling dates (2012 and 2014).
(XLSX)

S2 Table PLFA data. PLFA [nmol C g^-1^ DW] as affected by region (Hainich-Dün and Schorfheide-Chorin), forest type (young, old and unmanaged beech forests, coniferous forests), treatment (trenched and control subplots) and layer (litter and soil) at the two sampling dates (2012 and 2014).
(XLSX)

S3 Table Root tip data. Vital and mycorrhized root tips as affected by region (Hainich-Dün and Schorfheide-Chorin), forest type (young, old and unmanaged beech forests, coniferous forests) and treatment (trenched and control subplots) in 2014.
(XLSX)
